# The effect of cinnamon on polycystic ovary syndrome in a mouse model

**DOI:** 10.1186/s12958-018-0418-y

**Published:** 2018-10-19

**Authors:** Lei Dou, Yahong Zheng, Lu Li, Xiaowei Gui, Yajuan Chen, Meng Yu, Yi Guo

**Affiliations:** 1 Department of Obstetrics and gynecology, First Affiliated Hospital of China Medical University, Shenyang, 110001 China; 2 Department of Obstetrics and gynecology, Anshan Branch of First Affiliated Hospital of China Medical University, Anshan, China; 3 Department of Reproductive Biology and Transgenic Animals, China Medical University, Shenyang, China

**Keywords:** Cinnamon, Polycystic ovary syndrome, DHEA, Mice

## Abstract

**Background:**

Polycystic ovary syndrome (PCOS) is the most prevalent cause of anovulatory infertility and hyperandrogenism. Evidence favors insulin resistance and compensatory hyperinsulinemia as the predominant, perhaps primary, defects in PCOS. The use of insulin-sensitizing drugs has been shown to improve both the reproductive and the metabolic aspects of PCOS. Cinnamon has been found to have insulin sensitizing effect and improve menstrual cyclicity in women with PCOS. The aim of this study was to determine the effect and mechanism of cinnamon on PCOS using a dehydroepiandrosterone (DHEA) induced PCOS mouse model.

**Methods:**

Prepubertal C57BL/6 mice (age 25 days) were raised to developed into control group, DHEA group and DHEA plus cinnamon group for 20 days. The stages of the estrous cycle were determined based on vaginal cytology; metabolic characteristics were examined by intraperitoneal glucose tolerance test and insulin tolerance test, the serum levels of hormones (testosterone, insulin, LH, FSH, IGF-1, IGFBP-1) were checked using enzyme-linked immunosorbent assay (ELISA) method, the ovarian morphology was observed by stained with hematoxylin and eosin. IGF-1 and IGFBP-1 expression in ovary were detected by immunohistochemical stain.

**Results:**

Cinnamon restores the cyclicity and ovary morphology in PCOS mice model induced by DHEA. There are significant differences of serum level of total testosterone (0.033 ± 0.009 ng/ml), among control group, DHEA and cinnamon group (0.052 ± 0.011 ng/ml), and DHEA group (0.079 ± 0.015 ng/ml); There was an increasing tendency of serum FSH level from DHEA group (5.02 ± 0.31 ng/ml), DHEA and cinnamon group (5.81 ± 0.51 ng/ml), to control group (7.13 ± 0.74 ng/ml); and there was a decreasing trend of serum LH level from DHEA group (3.75 ± 0.57 ng/ml), DHEA and cinnamon group (1.35 ± 0.61 ng/ml), or control group (0.69 ± 0.34 ng/ml); serum insulin level is significantly higher in DHEA treated mice (1.61 ± 0.31 ng/ml) than control group (0.93 ± 0.19 ng/ml), or DHEA and cinnamon effect (1.27 ± 0.23 ng/ml) (*p* < 0.05). The DHEA group also has a higher serum IGF-1 level (0.35 ± 0.06 ng/ml) than control group (0.17 ± 0.04 ng/ml) or DHEA and cinnamon group (0.21 ± 0.05 ng/ml) (*p* < 0.05). While DHEA group has a lower IGFBP-1 level (5.5 ± 1.6 ng/ml) than control group (15.8 ± 2.1 ng/ml) or DHEA and cinnamon group (10.3 ± 2.5 ng/ml) (*p* < 0.05). Cinnamon also attenuates DHEA induced a higher IGF-1 and lower IGFBP-1 expression in ovary by immunohistochemistry.

**Conclusions:**

These preliminary data suggest that cinnamon supplementation improves insulin resistance and may be a potential therapeutic agent for the treatment of PCOS.

## Background

Polycystic ovary syndrome (PCOS) is one of the most common, complex and heterogeneous endocrine disorders affecting 5–10% of women of fertile age [1]. The manifestation of this syndrome includes oligomenhorrhea, amenorrhea, anovulation, numerous antral follicles, hypersecretion of circulating LH but with lower or equivalent FSH levels, hyperandrogenemia and hirsutism [2, 3]. Most of the patients also have metabolic abnormalities, such as obesity, dyslipidemia, and insulin resistance [4]. Women with PCOS are at increased risk of reproductive abnormalities, and two-thirds of them also have metabolic dysfunction and, thereby, have an increased risk of developing type 2 diabetes mellitus and cardiovascular disease [3]. The prevalence of insulin resistance and the compensatory hyperinsulinemia among women with PCOS is 50–70% [5] and may be as high as 95% in overweight women [6]. Hyperinsulinemia may promote abnormal androgen secretion and disrupt folliculogenesis and menstrual cyclicity which are the main characteristics of PCOS [7, 8].

Although the etiology of PCOS is still unclear, what we know is that genetic and environmental factors contribute to the origin and development of this disorder [9, 10]. Under such circumstances, Nutraceuticals may represent a valuable alternative or adjunct to lifestyle interventions and conventional prescription drugs. Cinnamon, which is a spice used to flavor foods, has been shown to possess anti-PCOS and anti-diabetic properties [11–13]. In a high-fructose diet induce insulin resistance rat model, cinnamon extract not only improves systemic insulin sensitivity and dyslipidemia by enhancing insulin signaling, but also effectively ameliorates circulating levels of adipokines partially mediated via regulation of the expression of multiple genes involved in insulin sensitivity and lipogenesis [14, 15]. Several in vitro and in vivo studies have shown cinnamon can reduce insulin resistance by increasing activation of the IRS/PI-3 kinase insulin signalling pathway [13].The extracts from cinnamon stimulate autophosphorylation of the insulin receptor and inhibit protein tyrosine phosphatase I [16]. Through these two mechanisms cinnamon extract make adipocytes to increase the glucose uptake and glycogen synthesis. Oral cinnamon extract reduced fasting glucose, triglycerides, low-density lipoprotein (LDL), and total cholesterol in patients with type 2 diabetes mellitus [17], as well as improved insulin sensitivity in women with PCOS [12]. Based on these findings, we put forward our hypothesis that cinnamon has an overall impact on PCOS treatment [11]. Previous studies proved dehydroepiandrosterone (DHEA) induced PCOS model represents similar characteristic seen in human patients, such as hyperandrogenism, abnormal maturation of ovarian follicles and anovulation [18, 19]. In this experiment, we tried to gain a deeper understanding of the effect and mechanism of cinnamon on PCOS using a DHEA induced PCOS like mice model.

## Methods

### Animals and experimental protocols

Sixty SPF grade female prepuberal C57BL/6 mice (21-day-old)were purchased from Beijing Vital River Laboratories animal co., LTD (license: SCXK(HU)2007–0003). All animals were raised in China Medical University Animal Center, 25 °C constant temperature (Humidity 50%), 12 h light:12 h dark cyclical alternates, with food and water available ad libitum. All procedures described here were reviewed and approved by the Ethical Committee of China Medical University. At Postnatal Day 25, these mice of comparable weights (12.89 ± 1.41 g)were randomly divided into three groups (control group, DHEA group, DHEA+cinnamon group) and treated for 20 consecutive days as followings (from the day 1 to the day 20). Control group (*n* = 10): The mice were injected subcutaneously daily with 0.1 ml sesame oil and 100 μL 0.5% methylcellulose given once daily using gavage needle. DHEA group (*n* = 25): The murine model of PCOS was developed by daily injecting DHEA (6 mg/100 g body weight dissolved in 0.1 ml of sesame oil) subcutaneously as described previously [20, 21] and 100 μL 0.5% methylcellulose given once daily using gavage needle. The DHEA+cinnamon group (*n* = 25): The mice were injected the same amount of DHEA while given cinnamon powder (10 mg/100 g body weight mixed in 100 μL 0.5% methylcellulose) via gavage as described [22]. After 20 days of the treatments, both reproductive and metabolic features were evaluated. The treatments were continued until the mice were killed. Throughout the whole treatment period, the animals were weighed every two days. All samples were run in triplicate.

### Estrous cycle determination

The vaginal smears were taken daily at 9 AM from the 13 days after the first injection (Day 38 of life) for 7 consecutive days. The stages of the estrous cycle were determined daily based on vaginal cytology [23]: Vaginal cells were collected via saline lavage and then fixed with methanol and stained with methylene blue staining (0.1%). Predominant nucleated epithelial cells and some cornified epithelial cells indicated the proestrus stage; predominant cornified squamous epithelial cells indicated the estrus stage; both cornified squamous epithelial cells and leukocytes indicated the metaestrus stage; and predominant leukocytes indicated the diestrus stage.

### Intraperitoneal glucose tolerance test

Right after 20 days of the treatment (Day 45 of life), Intraperitoneal(IP) glucose tolerance test (IPGTT) was performed [24]. The mice were fasted overnight (16 h) before the morning of the IPGTT. The mice were injected ip with glucose (2 g/kg as a 50% glucose stock solution). Glucose levels were measured by tail vein blood sampling using a blood glucose meter (Sinocare Inc., Changsha, China) immediately before the mice were injected intraperitoneally with glucose and then at 30, 60, 90, and 120 min after administration. Data were expressed as the absolute values of blood glucose concentrations. Total area under the curve of the glucose response (AUC) was calculated using GraphPad Prism 5.0 software.

### Insulin tolerance test

Five days after the IPGTT experiment, an insulin tolerance test (ITT) was carried out (Day 51 of life) as previously described [25]. The mice were fasted for 6 h and then injected intraperitoneally with insulin (1 IU/kg body weight, Wanbang Biopharmaceuticals, China). Blood glucose was measured immediately before insulin administration and then at 30, 60, 90, and 120 min after injection. Data were expressed as the values of blood glucose concentrations corrected for fasting glucose. Total AUC of the corrected glucose values was calculated using GraphPad Prism 5.0 software.

### Hormonal analysis of serum

After 21 days of treatment (47 days of life), the mice were anesthetized via an intraperitoneal injection of a mixture of 100 mg/ml Ketamine and 20 mg/ml Xylazine (Sigma-Aldrich Chemical, St. Louis, Missouri, USA) prepared in injectable saline (0.1 ml/10 g body weight) [26], then underwent a retrobulbar venous plexus blood draw after overnight fasting. Then the serum concentration level of testosterone, FSH, LH, IGF-1, IGFBP-1,and insulin were measured using by mouse ELISA Kit (R&D Systems. USA for testostereon testing, and Muyuan bio-tech company, Shanghai, China for the other hormones testing), as previously described [27]. All measurements were taken according to the manufacturer’s instructions. The detection limit was 0.041 ng/mL for testosterone, 0.032 ng/ml for FSH, 0.048 ng/ml for LH, 2.05 ng/ml for IGF-1 and 0.35 ng/ml for IGFBP-1. The intra- and inter-assay variation was 5.2% and 8.6% for testosterone, 3.1% and 5.4% for FSH, 5.6% and 6.3% for LH, 4.1% and 3.9% for IGF-1, 4.7% and 4.3% for IGFBP-1.

### Ovary collection and histology

At 47 days of life, immediately after the last blood draw, ovary samples were then rapidly removed from the animals. Ovaries from each mouse were fixed in 4% paraformaldehyde (PFA) and postfixed in 20% sucrose solution. Afterward, tissues were embedded into Tissue-Tek (O.C.T. compound; Sakura), frozen at − 80 °C overnight, and cut into sections (6-μm thickness) with a Leica Cryostat (LEICA CM1850). The ovary sections were stained with hematoxylin and eosin (H&E) according to the standard histological procedures. To examine ovarian morphology, every 12th section was mounted on a glass slide. Numbers of corpora lutea and large antral follicles were counted [28, 29]. The follicle diameter was calculated as the mean distance between opposite basal membrane portions, while the wall thickness was calculated as the sum of theca interna and granulosa cell layers. A cystic follicle is considered to be a large fluid filled cyst with an attenuated granulosa cell layer and thickened theca cell layer [30]. The count of large antral follicles was performed based on the mean diameter of the follicles greater than 300 μm. The area of the largest follicles was measured with ImageJ (National Institutes of Health) software.

### Immunohistochemistry

Ovarian sections were prepared as described above and Immunohistochemistry was performed as described previously [31]. Sections (5 μm thickness) were deparaffinized and permeabilized with 0.5% triton X-100 in tris buffered saline (TBS) for 10 min followed by 2 washes with TBS. Endogenous peroxidase activity was quenched with 3% (vol/vol) H_2_O_2_ in methanol for 15 min. Antigen retrieval was performed by heating sections in 10 mM citrate buffer. Tissue sections were blocked with 1% bovine serum albumin for 20 min at 37 °C, followed by overnight incubation at room temperature with the goat anti mouse IGF-1 polyclonal antibody (1:40; AF-291-NA, R&D, USA), or anti-IGFBP-1 polyclonal antibody (1:40; AF871, R&D, USA). Nonimmune immunoglobulin G (IgG) was used as the negative control. Sections were then incubated with a polyperoxidase-conjugated rabbit anti-goat IgG (Zhongshan Golden Bridge) for 20 min at 37 °C. Immunostaining was revealed by using diaminobenzidine and counterstained with hematoxylin. Immunostaining intensity was evaluated by three independent observers not involved with the study who were asked to rate the intensity of the staining without knowing the identity of samples.

### Statistical analysis

Data are presented as mean ± SEM. Statistical analyses were performed with statistical software package SPSS. A One-way analysis of variance and least significant difference Tukey’s post hoc tests were used to evaluate the hormonal differences among the groups. A two-way analysis of variance with repeated measures for time was used to test significances of IPGTT and ITT experiment. Statistical significance was set at *P* < 0.05.

## Results

### Cinnamon restores estrous cyclicity and ovary morphology

**Fig. 1:**
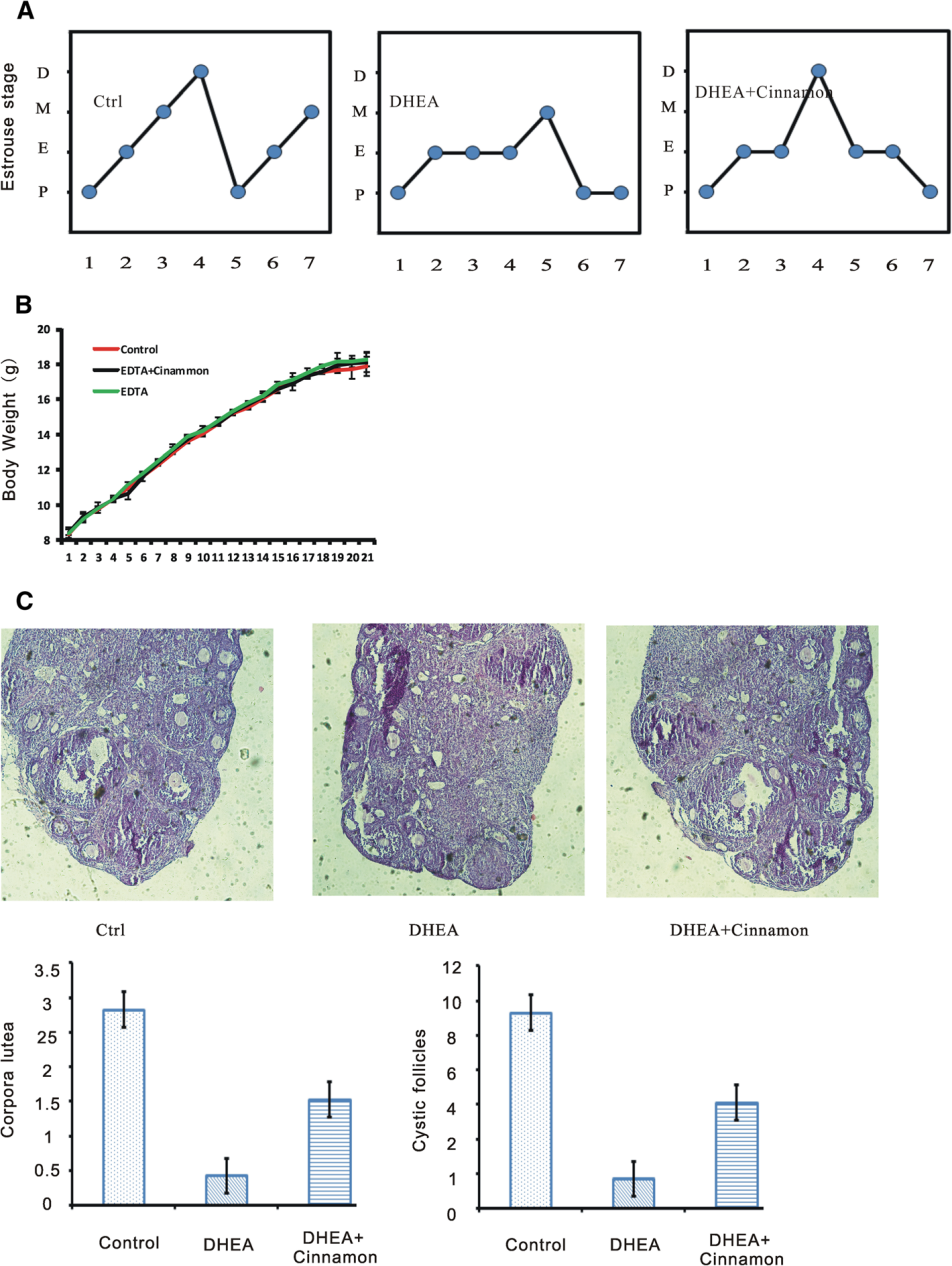
Estrous cycle, body weight, and ovarian morphology in the mice. **a** Representative estrous cycle of one mouse from each group. D,diestrus; M, metestrus; E, estrus; P, proestrus. **b** Body weight. **c** Representative H&E staining of ovarian sections of one mouse from each group. Micrographs were taken at magnifications× 200

As a result, all mice from DHEA group were completely acyclic and remained in constant estrus while control mice had normal cycle. The mice from DHEA plus cinnamon group have cycle but less than controls. Representative cyclicities of mice in three groups are showed in Fig. 1a. All of the mice (10 of 10,100%)in the control group showed normal estrous cyclity, while (20 of 25, 80%) the mice treated with DHEA displayed abnormal estrous cycles. Cinnamon treatment recover the disrupted estrous cycle induced by DHEA with a longer period (17 of 25, 68%). We did not find the difference of the weight of mice body among the three groups of mice as showed in Fig. 1b. Typical micrographs of ovarian sections of the three groups are shown in Fig. 1c. Comparing with DHEA plus Cinnamon or control group, DHEA treated mice exhibited a significant decrease of morphologically healthy large antral follicles, their identifying characteristic is a fluid-filled cavity and the oocyte lies at the edge in a mound made of granulosa epithelial cells [17]. The number of corpora lutea and oocyte in DHEA group was significantly lower than the DHEA plus Cinnamon or control group (*P* < 0.05). The thickness of the granulosa cell layer of large antral in DHEA group is less and the cells appear degenerated.

### Cinnamon effect on hormone level

**Fig. 2:**
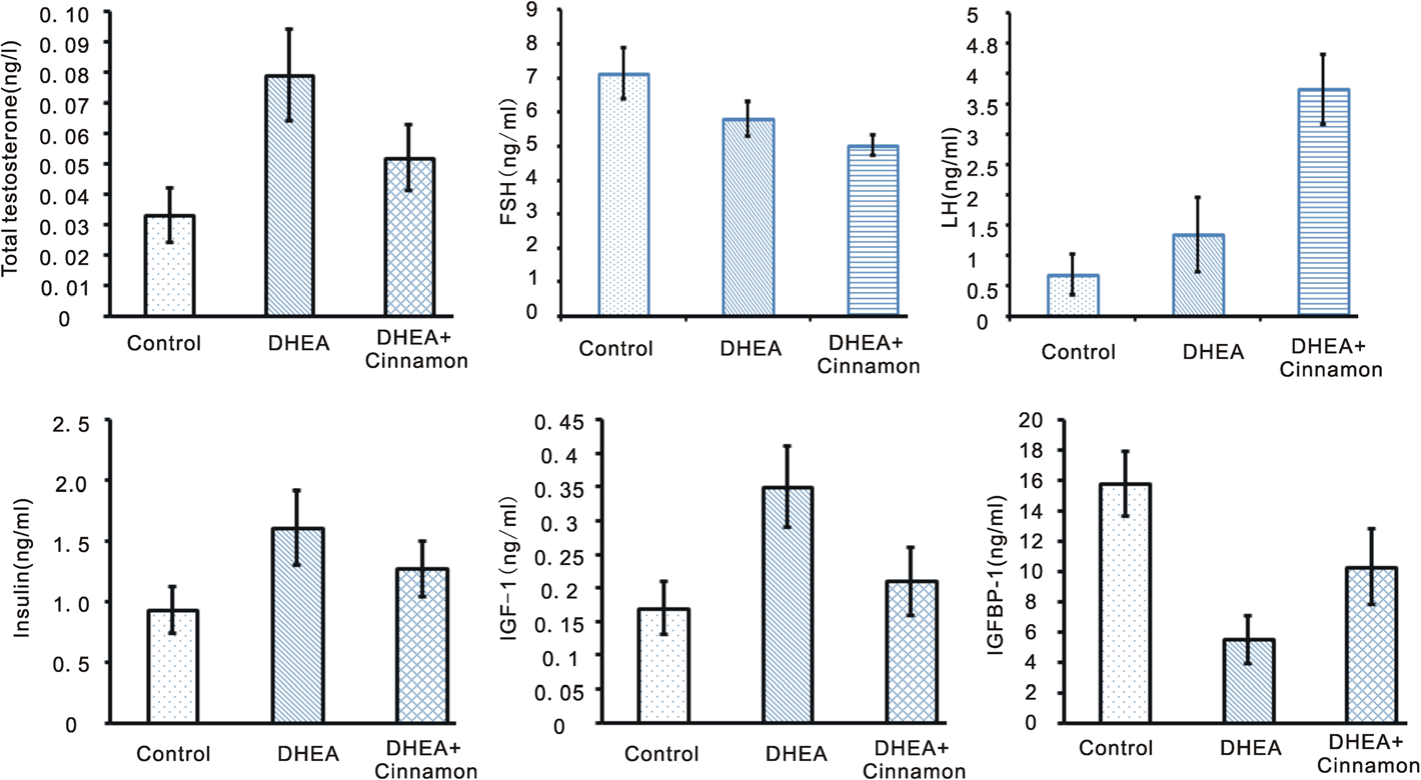
Serum levels of testosterone, insulin, FSH, LH, IGF-1 and IGFBP-1in the mice. Data are presented as mean ± SEM. *n* = 8 per group

Total testosterone is increasing from control group (0.033 ± 0.009 ng/ml), to DHEA and cinnamon group (0.052 ± 0.011 ng/ml), to DHEA group (0.079 ± 0.015 ng/ml), the differences among them were significant (*p* < 0.05). There was an increasing tendency of serum FSH level from DHEA group (5.02 ± 0.31 ng/ml), DHEA and cinnamon group (5.81 ± 0.51 ng/ml), to control group (7.13 ± 0.74 ng/ml); and there was a decreasing trend of serum LH level from DHEA group (3.75 ± 0.57 ng/ml), DHEA and cinnamon group (1.35 ± 0.61 ng/ml), or control group (0.69 ± 0.34 ng/ml); The differences of the ration of LH to FSH among control group, DHEA group and DHEA plus cinnamon group were significant (*p* < 0.05). Serum insulin level was significant higher in DHEA treated mice (1.61 ± 0.31 ng/ml) than control group (0.93 ± 0.19 ng/ml), add cinnamon can attenuated DHEA effect on insulin in the mice model (1.27 ± 0.23 ng/ml) (*p* < 0.05). The DHEA group also have a higher serum IGF-1 level (0.35 ± 0.06 ng/ml) than control group (0.17 ± 0.04 ng/ml) or DHEA and cinnamon group (0.21 ± 0.05 ng/ml) (*p* < 0.05). While DHEA group have a lower IGFBP-1 level (5.5 ± 1.6 ng/ml) than control group (15.8 ± 2.1 ng/ml) or DHEA and cinnamon group (10.3 ± 2.5 ng/ml) (*p* < 0.05); (Fig. 2).

### IPGTT and ITT experiments

**Fig. 3:**
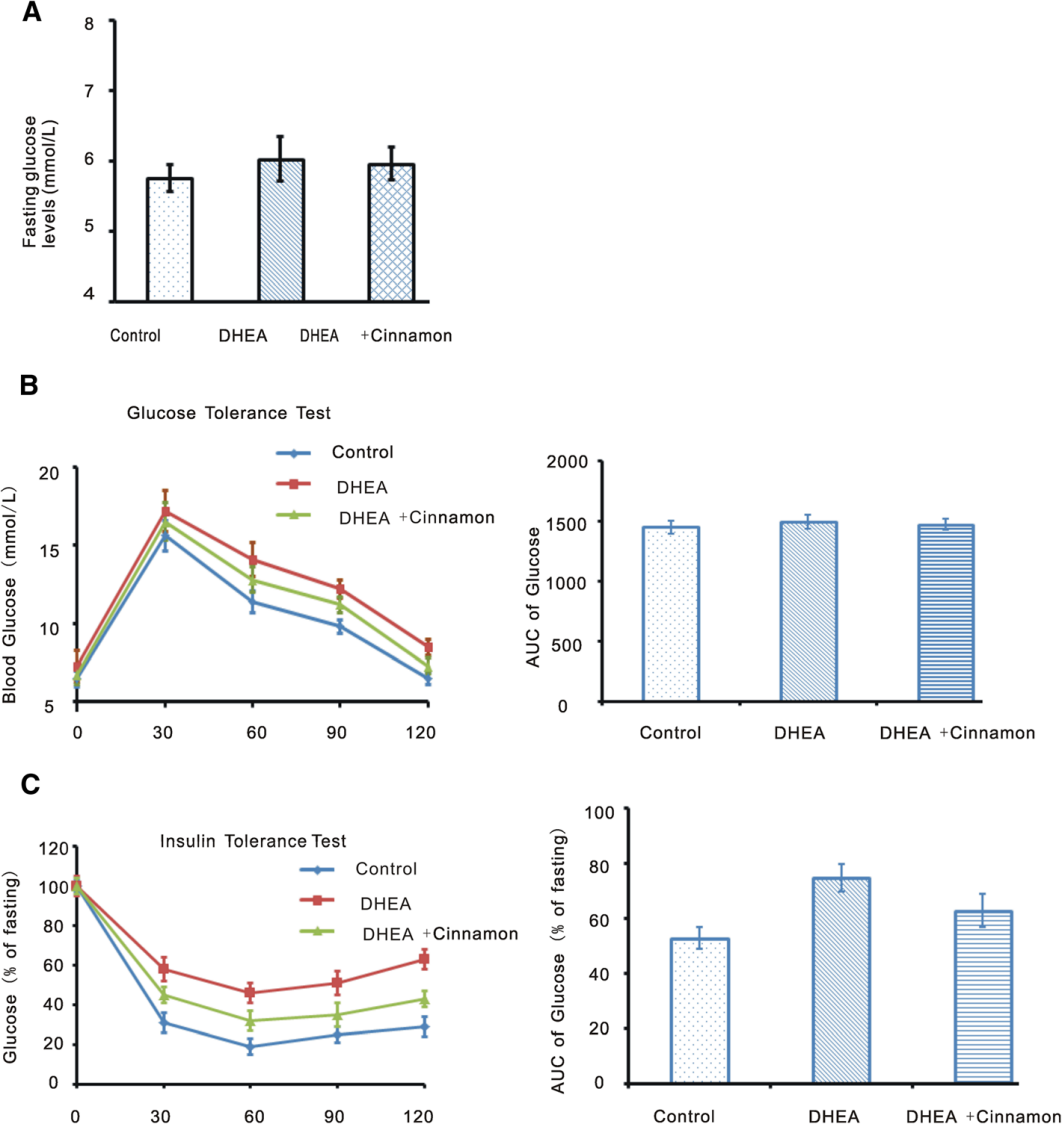
Fasting glucose, IPGTT and ITT Experiments in the mice. **a** Fasting glucose. **b** IPGTT experiment, IPGTT curve and IPGTT AUC. **c** ITT experiment, Serum glucose levels corrected for fasting glucose and the AUC of the corrected glucose levels. Data are presented as mean ± SEM. *N* = 8 per group

We performed IPGTT to investigate cinnamon effect on glucose tolerance of DHEA induced PCOS model. There were no differences of fasting glucose level among the three groups (Fig. 3a). Neither differences in 0- to 120-min AUC value existed among the tree groups (Fig. 3b). We also performed Insulin tolerance test. Blood glucose levels were corrected for fasting glucose. There were significant differences of corrected serum glucose levels among the three groups of mice after injection (*P* < 0.05). Correspondingly, the differences of AUC were also significant among the three groups (*P* < 0.05; Fig. 3c). In addition, these data suggested the impaired glucose tolerance in DHEA induced PCOS mice was likely be mitigated by cinnamon treatment.

### Cinnamon effects on IGF-1 and IGFBP-1 expression in ovary

**Fig. 4:**
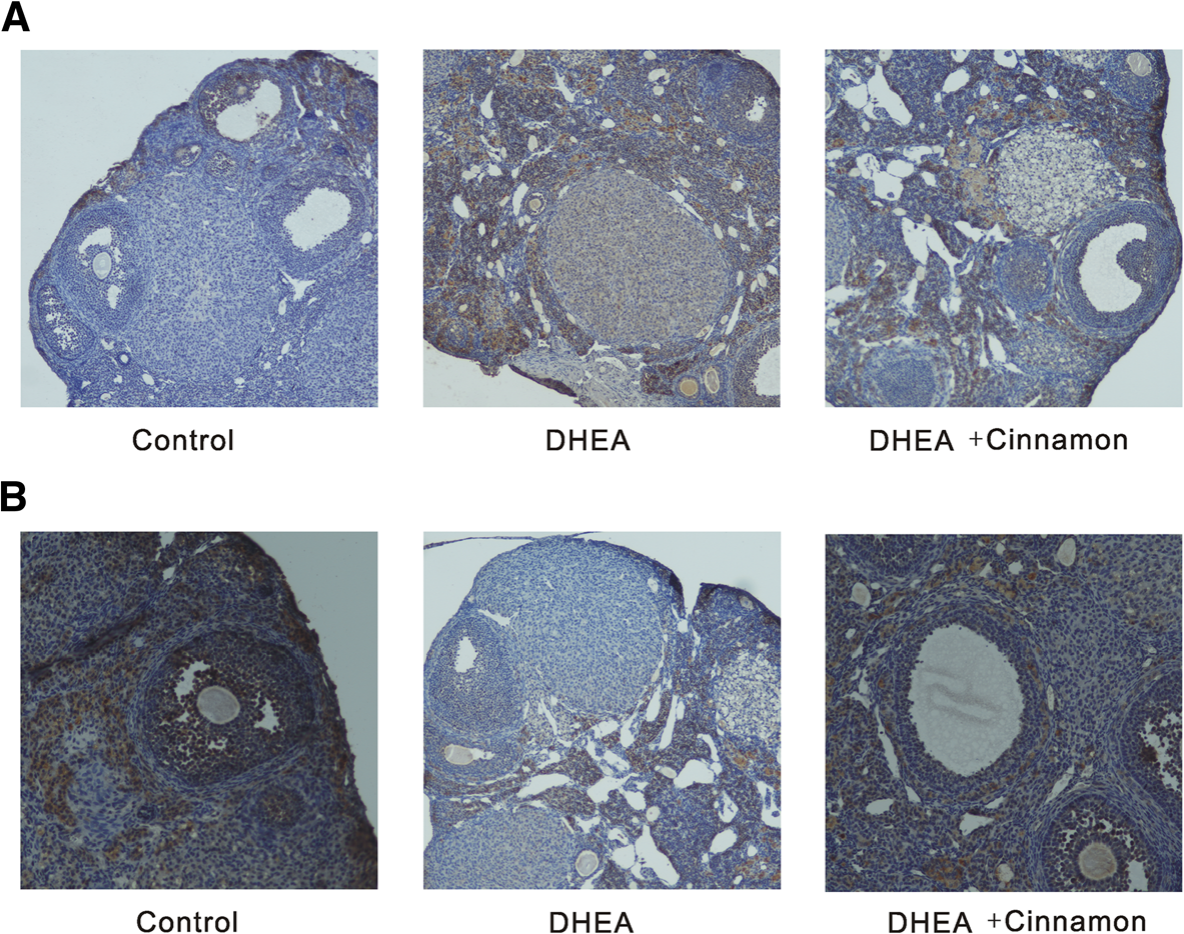
**a** Weak staining of IGF-1 in ovary of control mice group, strong staining of DHEA mice group, moderate staining of DHEA and cinnamon group. **b** Strong staining of IGFBP-1 in ovary of control mice group, weak staining of DHEA mice group, moderate staining of DHEA and cinnamon group

In the normal ovary of control group, there was weak IGF-1 staining was seen in oocyte and granulosa cell of small antral follicles. Atretic follicle and the surrounding stroma in DHEA treated group showed a strong IGF-1 staining. There was a moderate IGF-1 staining in DHEA and cinnamon group (Fig. 4a). While large follicles in the ovary of control mice showed a strong IGFBP-1 staining. Atretic follicles in the ovary of DHEA treated mice have no IGFBP-1 staining. Follicles in the ovary of DHEA and cinnamon treated mice have a moderate IGFBP-1 staining (Fig. 4b).

## Discussion

As a complex disorder consisting multiple phenotypic parameters, PCOS patients have neuroendocrine and ovarian impairments, as well as a high prevalence of metabolic perturbation, such as obesity and glucose intolerance [32, 33]. There is clear evidence that insulin stimulates ovarian thecal and stromal androgen secretion in vitro [34]. In women with PCOS, insulin resistance with compensatory hyperinsulinemia induces overproduction of ovarian androgens, leading to hyperandrogenism. The action of insulin on the production of androgens in the ovary is believed to be through IGF-1 receptors on theca and stroma cells [35, 36]. IGF-1 stimulates estrogen production by granulosa cell [37] and acts synergistically with FSH and LH in controlling granulosa cell aromatase concentration [38]. IGF-1 also acts synergistically with LH to stimulate androgen production on thecal cell [39]. High level of insulin and IGF-1 amplifies the effect of LH on granulosa cell, inducing terminal differentiation and leading to anovulation. IGF-1 and IGFBP-1affect follicular maturation by autocrine and/or paracrine mechanisms [40, 41]. It looks like that IGF-1 has negative impact on normal folliculogenesis and ovulation [39, 42]. Though total plasma IGF-1 level was not reported significantly higher in PCOS patients [43], there is a decreased plasma IGFBP-1 level in PCOS patients compared with normal women [44]. The reduction of IGFBP-1 concentration may raise IGF-1 bioavailability, in fact, the free plasma IGF-1 concentration is higher in PCOS patients [45].

Insulin resistance likely contributes to the hallmark symptoms of PCOS such as androgen excess and menstrual irregularity, as well as increases the risk of developing diabetes mellitus, hyperlipidemia and cardiovascular diseases [46]. Hence, reducing insulin resistance not only provides potential reproductive health benefits but more importantly is the key to improving the overall long-term health of patients with PCOS.

Cinnamon, a commonly used spice and flavoring material, has a long history as a medicine as well. The cinnamon has been recognized as insulin potentiating factor almost 20 years ago [47]. Cinnamon extracts act as enhancer of enhancer of insulin-receptor function and inhibitor of the enzyme that blocks insulin-receptor attachment, respectively. Procyanidin polyphenol type-A polymers extracted from cinnamon and has the ability to stimulate autophosphorylation of the insulin receptor and inhibit protein tyrosine phosphatase I. Insulin receptor kinase autophosphorylation and subsequent phosphorylation of its principal substrate are markedly decreased in insulin-responsive tissues of subjects with severe obesity or non-insulin-dependent diabetes mellitus (NIDDM) [48]. The inhibition of tyrosine phosphatase by cinnamon would prove that insulin resistance may be due to elevated tyrosine phosphatase activity [49]. Cinnamon has been showed to have anti-diabetes and anti-obese properties. Cinnamon extract has been found to mitigate insulin resistance induced by high fructose diets [14] and benefit glucose utilization by enhancing insulin signaling pathway [15]. Cinnamon extract increases glycogen synthesis by activating glycogen synthase and inhibiting glycogen synthase kinase 3β [50], and reduces glucose absorption in the small intestine through increasing in glucosidase enzymes and inhibition of intestinal ATPase [51, 52]. Cinnamon also contains other polyphenolic compounds like rutin, catechin, quercetin and kaempferol which have insulin like activity [53]. In a study to explore the mechanism of cinnamon on glucose regulating, cinnamic acid with 5 and 10 mg/kg doses administered orally to diabetic rats improved glucose tolerance in a dose-dependent manner; as well as in vitro studies showed that cinnamic acid significantly enhanced glucose-stimulated insulin secretion in isolated islets [54]. So cinnamic acid exerts the function of improving glucose tolerance in vivo and stimulating insulin secretion in vitro. Cinnamaldehyde, one of the active components of cinnamon, has been tested for its antiobesity by investigating the antidifferentiation effect on 3 T3-L1 preadipocytes, and high-fat-diet-induced obese ICR mice. It was found that cinnamaldehyde significantly reduced lipid accumulation and down-regulated the expression of peroxisome proliferator-activated receptor-γ (PPAR-γ), CCAAT/enhancer-binding proteins α (C/EBPα), and sterol regulatory element-binding protein 1 (SREBP1) in concentration-dependent manners. Moreover, cinnamaldehyde markedly up-regulated AMP-activated protein kinase (AMPK) and acetyl-CoA carboxylase (ACC), and these effects were blunted in the presence of AMPK inhibitor, compound C [55, 56]. In the animal study, weight gains, insulin resistance index, plasma triglyceride (TG), nonesterified fatty acid (NEFA), and cholesterol levels in the 40 mg/kg of cinnamaldehyde-administered group were significantly decreased by 67.3, 55, 39, 31, and 23%, respectively, when compared to the high-fat diet control group [55]. Cinnamon can also modulate the insulin and IGF1 signaling pathways such as mTOR, Cyclic-AMP signaling and autophagy [57].

As a folk medicine, cinnamon shows hepatoprotective [58], anti-oxidant [59], anti-obesity [60], antihyperlipidemic [61], and antidiabetic activities [62]. A randomized controlled clinical trial has provided evidence that cinnamon supplementation improves menstrual cyclicity and may be an effective treatment option for some women with PCOS [11]. The underlying mechanism for cinnamon effects on PCOS may contribute to its improvement in insulin sensitivity. Insulin receptor is the entry of insulin signaling pathway that mediates the pleiotropic actions of insulin. Insulin receptor substrate (IRS) proteins act as docking molecules to connect tyrosine kinase receptor activation to essential downstream kinase cascades, including activation of the PI-3 kinase or MAPK cascade [63]. Phosphorylation of IRS proteins leads to the activation of these two main signaling pathways. The PI-3 kinase pathway is responsible for most of the metabolic actions of insulin, and the MAPK pathway regulates expression of some genes and cooperates with the PI-3 kinase pathway to control cell growth and differentiation. The expression level of IRS protein has been found to be significantly downregulated in the model animals and upregulated after cinnamon treatment [64]. In the first randomized, controlled trial, cinnamon at different dosages (1 g, 3 g, 6 g) decreased mean fasting glucose, triglycerides, LDL cholesterol, and total cholesterol in patients with type 2 diabetes [17]. A prospective placebo-controlled pilot study showed that cinnamon demonstrated significant reductions in fasting glucose and insulin resistance parameters after 8 weeks of oral cinnamon extract 1 g per day [12].

Different kinds of Rodent models have been developed to study the mechanisms and treatment effects of PCOS [65, 66]. DHEA, as the most abundant steroid hormone in the circulation, has been used to induce a PCOS mouse model for almost a decade [20]. The DHEA induced PCOS mice model has disturbed cyclicity, multicystic ovaries, and hyperandrogenism.

In this study, we showed that oral administration of cinnamon extract would restore cyclicity, down-regulate testosterone and improve insulin sensitivity in DHEA-induced PCOS mice with the C57BL/6 background.

The results of this study confirmed the effects that cinnamon had on the reduction of insulin resistance. Although the effects of cinnamon on diabetes and its related blood parameters and lipid profile have been studied in several trials, to our knowledge, this is the first study indicating the therapeutic effects of cinnamon on PCOS characteristics with animal model. In this study, we confirmed cinnamon effect in treating PCOS by reducing the level of IGF-I and increase the level of IGFBP-1 in plasma as well as in ovary tissue.

## Conclusions

In summary, our study demonstrated that cinnamon had the ability to restore the estrous cyclicity and ovary morphology, down-regulate serum levels of testosterone and insulin, decrease IGF-1 level while increase IGFBP-1 level in plasma as well as in the ovary in DHEA induce PCOS mice model. Cinnamon may be a potential therapeutic agent for the treatment of PCOS.

## Abbreviations

AUC: Area under the curve
DHEA: Dehydroepiandrosterone
ELISA: Enzyme-linked immunosorbent assay
FSH: Follicle-stimulating hormone
HE: Hematoxylin-eosin
IGF-1: Insulin-like growth factor I
IGFBP-1: Insulin like growth factor binding protein-1
IHC: Immunohistochemical
IPGTT: Intraperitoneal Glucose Tolerance Test
IRS: Insulin receptor substrate
IRS/PI: Insulin receptor substrate/Phosphoinositide
ITT: Insulin tolerance test
LDL: Low-density lipoprotein
LH: Luteinizing hormone
MAPK: Mitogen-activated protein kinase
MC: Methylcellulose
NIDDM: Non-insulin-dependent diabetes mellitus
PCOS: Polycystic ovary syndrome
SPF: Specific pathogen Free
TBS: Tris buffered saline
